# Arthrosurface of the humeral head with the OVO surface replacement endoprosthesis

**DOI:** 10.1093/jscr/rjae794

**Published:** 2024-12-18

**Authors:** Maciej Wrotniak, Łukasz Polczak, Łukasz Skowron, Piotr Rydel, Adrian Urbanek, Marcin Kostuj

**Affiliations:** Clinical Department of Orthopedics, Plac Medyków 1, 41-214 Sosnowiec, Silesia, Faculty of Medical Sciences in Zabrze, Medical University of Silesia, Poniatowskiego 15, 40-055 Katowice, Silesia, Poland; Clinical Department of Orthopedics, Plac Medyków 1, 41-214 Sosnowiec, Silesia, Faculty of Medical Sciences in Zabrze, Medical University of Silesia, Poniatowskiego 15, 40-055 Katowice, Silesia, Poland; Clinical Department of Orthopedics, Plac Medyków 1, 41-214 Sosnowiec, Silesia, Faculty of Medical Sciences in Zabrze, Medical University of Silesia, Poniatowskiego 15, 40-055 Katowice, Silesia, Poland; Clinical Department of Orthopedics, Plac Medyków 1, 41-214 Sosnowiec, Silesia, Faculty of Medical Sciences in Zabrze, Medical University of Silesia, Poniatowskiego 15, 40-055 Katowice, Silesia, Poland; Clinical Department of Orthopedics, Plac Medyków 1, 41-214 Sosnowiec, Silesia, Faculty of Medical Sciences in Zabrze, Medical University of Silesia, Poniatowskiego 15, 40-055 Katowice, Silesia, Poland; Clinical Department of Orthopedics, Plac Medyków 1, 41-214 Sosnowiec, Silesia, Faculty of Medical Sciences in Zabrze, Medical University of Silesia, Poniatowskiego 15, 40-055 Katowice, Silesia, Poland

**Keywords:** arthrosurface, surface replacement endoprosthesis, fracture of the humeral head

## Abstract

The case report concerns a 67-year-old man who suffered an accident on a motorcycle, as a result of which he suffered a dented fracture of the right humeral head. After physical examination and imaging diagnostics, he was qualified for surgical treatment—arthrosurface of the patient’s humeral head with the OVO surface replacement endoprosthesis. Right after the patient’s surgical treatment, a period of 16-week rehabilitation was implemented, which, as a result, enabled the patient to achieve a full range of shoulder mobility, and the patient was able to return to all activities before the injury.

## Introduction

Fractures in the proximal end of the humerus have always been considered as a complicated case in terms of applied treatment due to the ambiguities that may occur in the diagnosis and qualification processes.

One of the basic problems of treating these fractures includes the decision between applying conservative or surgical treatment, which considers the type of fracture, additional damage, the structure of bone tissue, the patient’s biological age, life activity, and expectations. Unfortunately, statistically speaking, nonsurgical treatment often does not bring the expected results. On the other hand, surgical treatment may consist of open or closed repositioning of the fragments and their stabilization, which is crucial for the early initiation of the rehabilitation process. However, the difficulties result from the possibility of serious vascular-nerve complications because of intraoperative damage to soft tissues and also due to the technical difficulties of performing the reposition itself and maintaining it with the use of an appropriate implant, until bone regrowth is obtained.

Therefore, shoulder arthroplasty is becoming an increasingly common method of treating fractures of the proximal end of the humerus.

## Case study

Patient is a 67-year-old male who participated in a road accident on 28 April 2020. As a result of the accident, the motorcycle driver suffered a direct injury to his right shoulder. Immediately after this event, the patient was transported, diagnosed, and treated in the Emergency Ward, where shoulder contusion was diagnosed.

**Figure 1:**
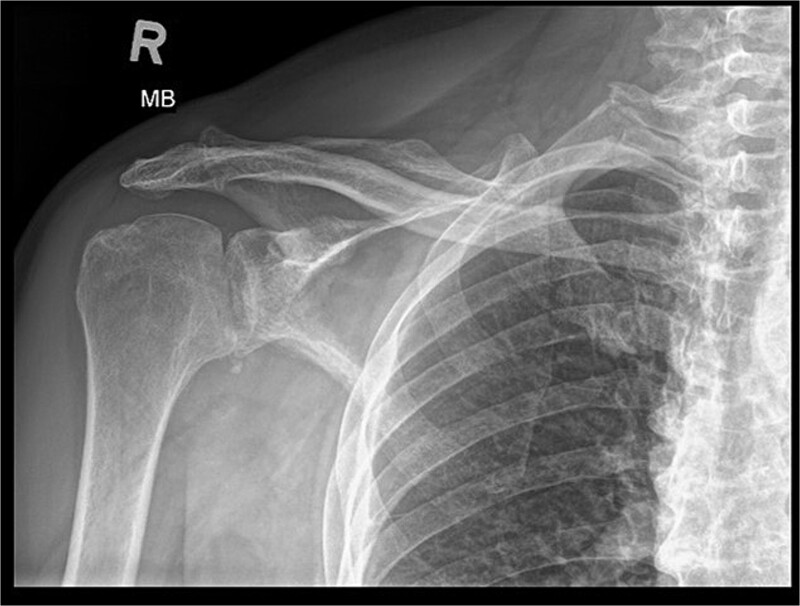
X-ray image before the surgery—AP projection.

**Figure 2:**
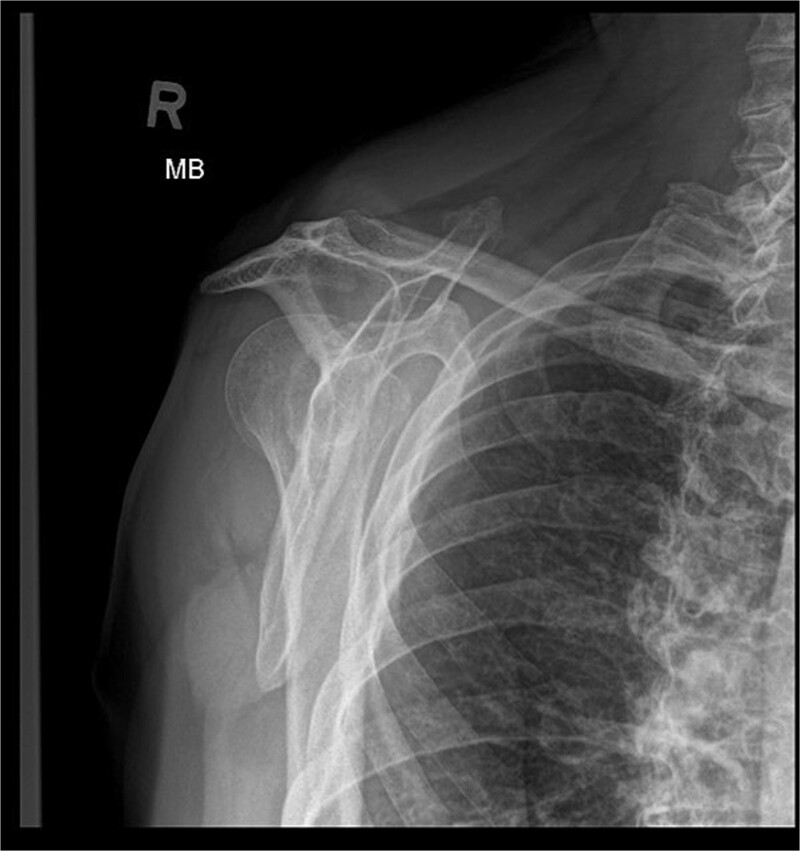
X-ray image before the surgery—Y projection.

X-ray images can be found in Figs 1 and 2.

After the diagnosis at EW, the decision was made to pursue with the conservative treatment.

During the outpatient follow-up visit at the Orthopedic Clinic, ~10 weeks after the accident, a physical examination of the patient was conducted, which included shoulder X-ray imaging. The examination revealed severe pain in the right shoulder and a significant limitation in the mobility of the joint (results summarized in Table 1). The above enforced the decision of expanding the imaging diagnostics to look for fractures within the humeral head; hence, computed tomography (CT) and magnetic resonance imaging (MRI) examinations of the right shoulder were carried out. Additional imaging studies revealed a depression fracture of the humeral head covering 2/3 of the articular surface with posterior subluxation of the right shoulder joint. The examination also revealed that the continuity of the tendons of the rotator cuff muscles has been preserved.

**Table 1:** Results of the range of motion of the shoulder joint. Range of motion was increasing quite dynamically in all planes.

	Motion	Before surgery		6 weeks after surgery		16 weeks after surgery	
	Range of motion	Active range of motion	Passive range of motion	Active range of motion	Passive range of motion	Active range of motion	Passive range of motion
1	Flexion	10	10	90	100	140	140
2	Extension	10	20	30	40	40	50
3	Abduction	20	30	90	110	170	170
4	Adduction	0	0	0	0	0	0
5	Internal rotation	15	20	40	45	55	60
6	External rotation	0	20	30	50	60	60
	**SFTR—active**						
	S	10-0-10		90-0-30		140-0-40	
	F	20-0-0		90-0-0		170-0-0	
	T						
	R (F0)	0-0-15		30-0-40		60-0-55	

**Figure 3:**
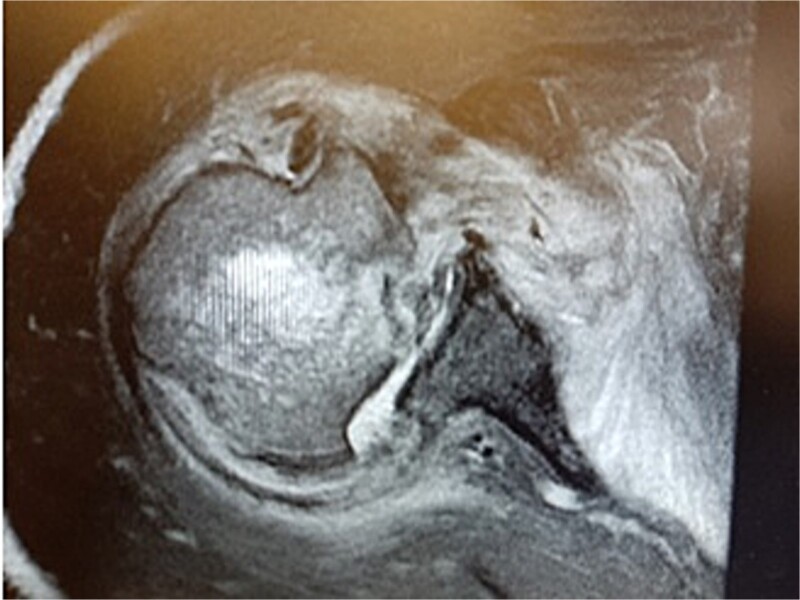
MRI image.

**Figure 4:**
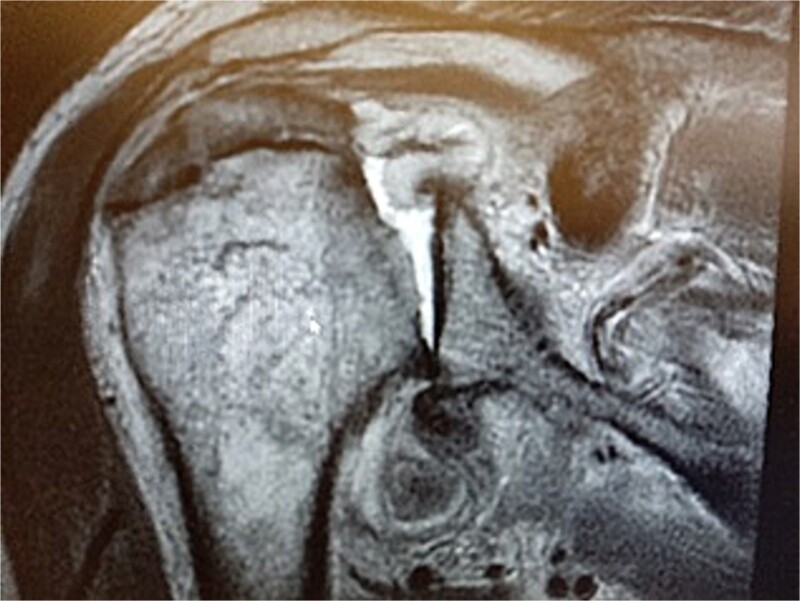
MRI image.

**Figure 5:**
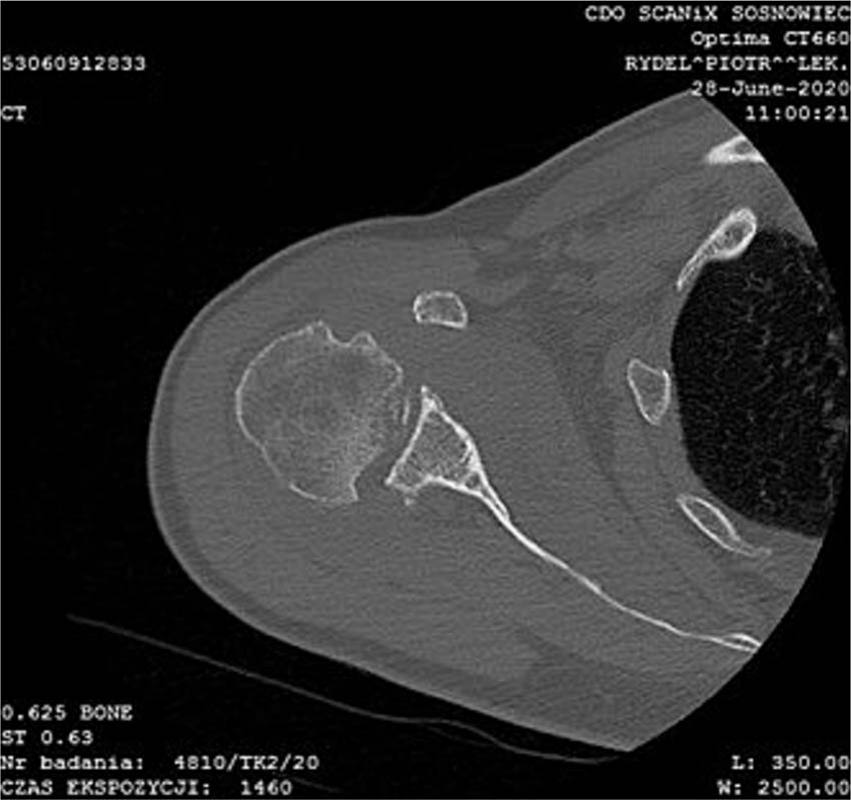
CT image.

MRI images can be found in Figs 3 and 4, and CT images in Fig. 5.

The lack of sufficient effects of the applied conservative treatment resulted in the patient being qualified for the orthopedic surgery.

On 17 December 2020, an arthrosurface procedure on the humeral head was performed (i.e. replacement of the damaged articular surface with an implant that recreates its desired shape) using the OVO Motion Shoulder Arthroplasty System Med & Care. The surgery was performed under general endotracheal anesthesia. During the surgery, the beach chair position of the patient with delto-pectoral approach was performed. The arthrotomy was performed by temporarily dissecting the tendon of the subscapular muscle. Additionally, an intraoperative SLAP lesion was found, and the tenodesis of the long head of the biceps muscle was performed. The operation was performed according to the standard surgical procedures during which an endoprosthesis of the humerus head articular surface (OVO) (diameter 48 × 52 mm) was implanted and cemented.

**Figure 6:**
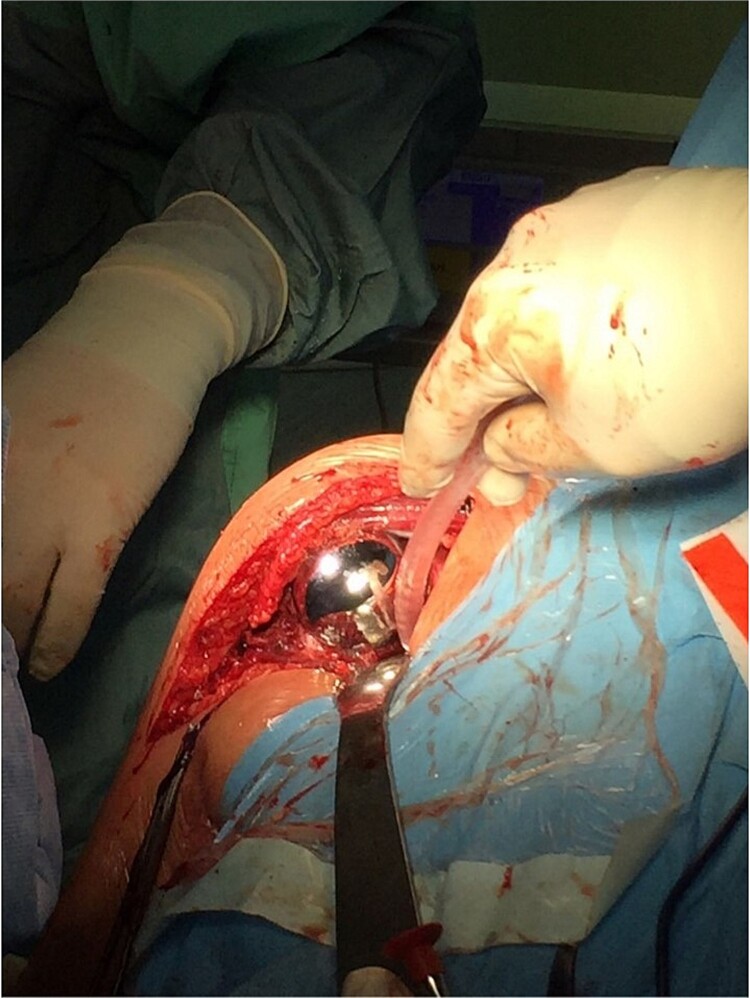
Intraoperative photo.

**Figure 7:**
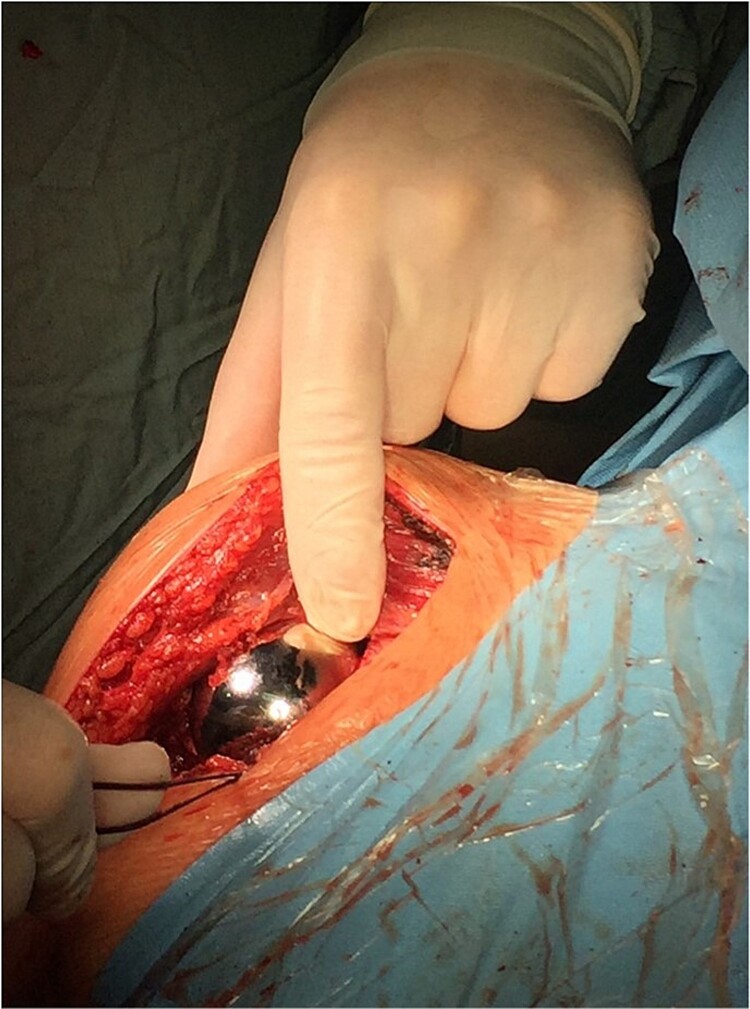
Intraoperative photo.

**Figure 8:**
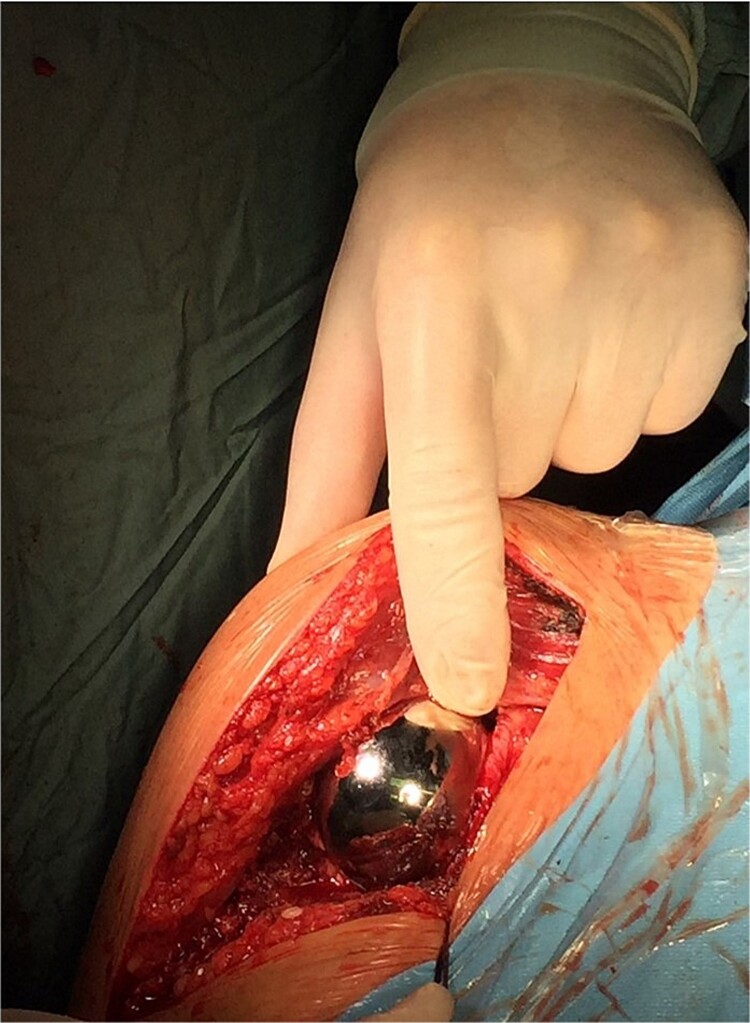
Intraoperative photo.

The operation procedure took ~1 hr. Below are intraoperative photos after the endoprosthesis has been placed (Figs 6–8).

**Figure 9:**
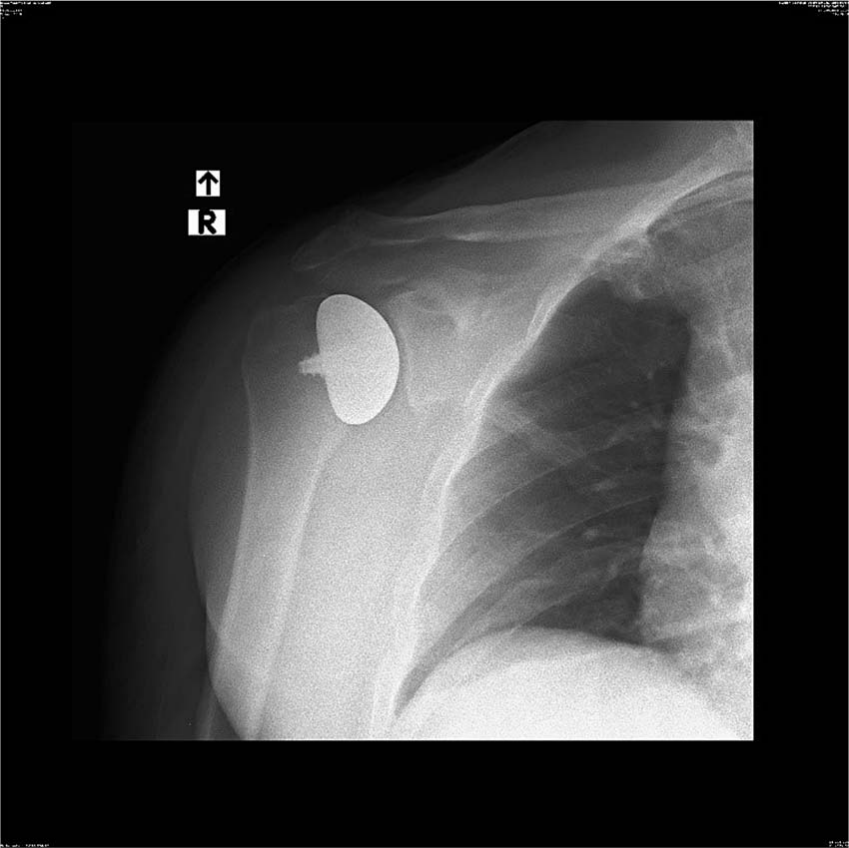
X-ray image after the surgery.

The peri and postoperative period passed without any complications. On day one after surgery, an X-ray image was taken (Fig. 9). The patient in good general and local condition was discharged from the hospital with a set of recommendations for rehabilitation. A sling was used to relieve the operated limb.

Rehabilitation was carried out from the day of the surgery and lasted for 16 weeks.

**Figure 10:**
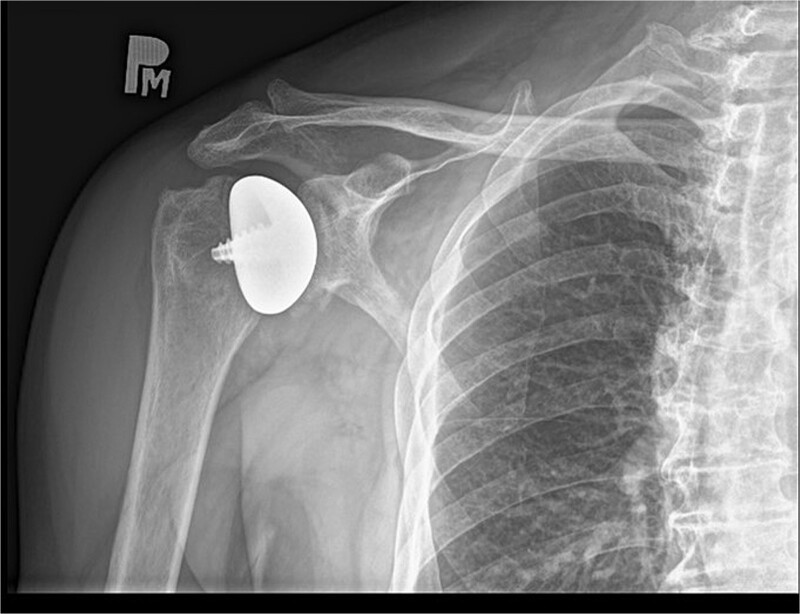
X-ray image after 16 days from the surgery.

As a result, almost the full range of motion with a significant improvement in muscle strength of the right shoulder joint was achieved without any relevant pain reported by the patient. The results were confirmed by another right shoulder X-ray image (Fig. 10). The patient has been instructed to resume his pre-injury regular activities, including lifting heavy objects.

## Results

For the needs of the patient’s physical condition assessment, the following were used: range of motion, muscle strength, and a functional scale. Detailed measurement results are presented in Tables 1–3.

**Table 2:** Medical Research Council (MRC) subjective 6-point scale for muscle strength assessment-results demonstrate significant increase of all muscle groups, in selected time points.

	Motion	Before surgery	6 weeks after surgery	16 weeks after surgery
1	Flexion	−4	3	−5
2	Extension	4	4	5
3	Abduction	−4	3	−5
4	Adduction	5	4	5
5	Internal rotation	4	4	5
6	External rotation	3	3	4
7	Horizontal flexion	Impossible to perform	3	4
8	Horizontal extension	Impossible to perform	3	4

**Table 3:** The Disabilities of the Arm, Shoulder and Hand (DASH) questionnaire for the right limb was conducted five times. The results show, that after 16 weeks from the treatment, the functional limitations of the injured limb are minor.

	Before surgery	2 weeks	6 weeks	10 weeks	16 weeks
1	4	5	3	2	1
2	1	3	1	1	1
3	2	3	2	1	1
4	4	4	3	2	1
5	4	4	4	3	1
6	5	5	4	3	2
7	4	5	3	2	1
8	3	4	3	3	2
9	4	4	2	1	1
10	4	5	3	2	1
11	4	5	4	2	1
12	5	5	5	4	2
13	4	5	4	2	1
14	4	5	4	3	2
15	4	5	3	2	1
16	3	3	2	1	1
17	2	3	2	1	1
18	5	5	4	3	1
19	5	5	4	4	2
20	2	3	2	1	1
21	3	4	2	1	1
22	4	4	3	2	1
23	4	5	4	3	1
24	3	4	3	2	1
25	4	5	4	2	1
26	3	3	2	1	1
27	4	5	4	2	1
28	3	5	4	2	1
29	4	5	3	1	2
30	5	5	4	3	2
DASH	66,66	84,16	54,16	26,66	5,83

## Discussion and summary

According to [1, 2], arthrosurface is considered as the least invasive shoulder replacement. The main condition for making a surface endoprosthesis in the shoulder is the lack of continuity of the tendons of the rotator cuff muscles or repairable damage that can be repaired during surgery [1, 2]. The main indications for the implementation of a shoulder surface endoprosthesis are defects in the articular cartilage and bone tissue of the humeral head and/or the acetabulum of the scapula of degenerative or post-traumatic origin [1–4]. A frequent indication for this type of endoprosthesis is avascular necrosis of the humeral head (AVN) and Hill Sachs lesion [5–7]. The chemical composition of the OVO implant is Co–Cr–Mo and titanium [5].

Other types of shoulder replacement are anatomical or reverse shoulder replacement. However, the implantation of these endoprostheses is far more invasive compared to OVO [3, 4, 6].

The main advantages of the OVO endoprosthesis over the other types of shoulder replacement are the fact that its shape best reproduces the geometry of the humerus head [1, 2, 5], as well as largely preserves bone tissue, which gives enhanced possibilities for potential revision surgery in the future [1–3, 5]. Due to the shorter surgery time and less blood loss, implanting OVO is the least invasive procedure [1, 2, 5], compared to other surgeries implanting endoprostheses. Further advantages are that the patient experiences less pain and can be discharged from the hospital quicker to start the rehabilitation process. After such surgery, it is possible to return to the activity from before the injury (including sports, such as weightlifting) [4, 7].

A surface replacement endoprosthesis should not be implanted in the case of irreparable damage to the rotator cuff [8–10] and in the case of having large defects of the humeral head, including the greater or lesser tubercle of the humerus [8–10].
